# Risk factors for mumps in children under 15 years of age during the transition from single-dose to two-dose MMR vaccination strategy: a matched case-control study

**DOI:** 10.3389/fpubh.2025.1592602

**Published:** 2025-07-07

**Authors:** Huaxian Liu, Yan Xie, Zhongkui Zhu, Yuanbao Liu, Yang Yu, Jintao Wang, Lu Shen, Yunke Qian, Wanqin Tang

**Affiliations:** ^1^Department of Expanded Programme on Immunization, Taizhou City Center for Disease Control and Prevention, Taizhou, China; ^2^Department of Expanded Programme on Immunization, Jiangsu Provincial Center for Disease Control and Prevention, Nanjing, China

**Keywords:** mumps, risk factors, children under 15 years of age, MMR vaccination strategy, matched case-control study

## Abstract

**Background:**

This study aimed to investigate the risk factors for mumps in children under 15 years of age during the transition from a single-dose to a two-dose measles–mumps–rubella (MMR) vaccine strategy, providing a scientific basis for public health policies and interventions.

**Methods:**

From the China Disease Prevention and Control (CDC) Information System, 547 children aged 0–14 years diagnosed with mumps in Taizhou City between 2021 and 2023 were included as cases. Controls were matched 1:1 and surveyed by the same investigator.

**Results:**

The analysis included 547 matched case-control pairs (343 males and 204 females) of children aged <15 years. The median age of the matched case-control pairs was 72 months. Among cases, children aged 49–72 months accounted for the highest proportion (28.52%). The multifactorial study showed that longer local residence (OR = 0.548, 95% CI 0.403 ~ 0.744), history of Mumps Component Vaccine (MuCV) (OR = 0.103, 95% CI 0.036 ~ 0.297), and belief that children would not get mumps (OR = 0.197, 95% CI 0.121 ~ 0.319) reduced the risk of mumps infection. In contrast, families with multiple births (OR = 1.926, 95% CI 1.405 ~ 2.640), being cared by someone other than parents (e.g., grandparents, babysitters, relatives or staff at a childcare center) (OR = 4.366, 95% CI 2.417 ~ 7.888), a higher level of the most frequently visited hospital (OR = 2.012, 95% CI 1.490 ~ 2.716), going into a crowded indoor place without wearing a mask (OR = 1.699, 95% CI 1.237 ~ 2.334), believing that mumps is not an infectious disease (OR = 1.782, 95% CI 1.274 ~ 2.492), believing that the disease is not serious (OR = 1.507, 95% CI 1.260 ~ 1.802), and believing that MuCV cannot prevent mumps (OR = 2.052, 95% CI 1.451 ~ 2.901) increased the risk of mumps infection.

**Conclusion:**

For mumps prevention and control, targeted interventions should prioritize children aged 4–6 years and high-risk populations, including short-term residents, multi-child families, and childcare settings. Guardians should be encouraged to gain accurate knowledge about mumps and the protective effects of vaccination to mitigate vaccine hesitancy, ultimately controlling mumps outbreaks in the population.

## 1 Introduction

Mumps is just an acute respiratory infection caused by the mumps virus (MuV), which is highly contagious and people of all ages are generally susceptible. The main clinical manifestations are painful swelling and non-suppurative inflammation of the parotid gland, which can easily lead to complications such as viral encephalitis, pancreatitis and orchitis (1). MuCV is the most effective strategy for the prevention and control of mumps (2). Since the MuV vaccine strain was introduced globally in 1966, the number of mumps cases has decreased significantly, and with the promotion of the implementation of the two-dose measles–mumps–rubella (MMR) vaccination strategy in 1989, the number of mumps cases has fallen to an all-time low (3). In countries achieving high coverage of two-dose MMR vaccination, mumps incidence has plummeted from 100–1,000 to <1 case per 100,000 population, and Finland has already become the first country in the world to eliminate mumps and rubella by implementing a two-dose MMR vaccination program (1 dose at 14–18 months of age and 1 dose at 6 years of age) (4). In most countries, the epidemiological cycle of mumps usually occurs every 2–5 years (5), and large-scale outbreaks of mumps have been observed in the United States, Norway, and other countries since 2015 (6, 7).

**Figure 1:**
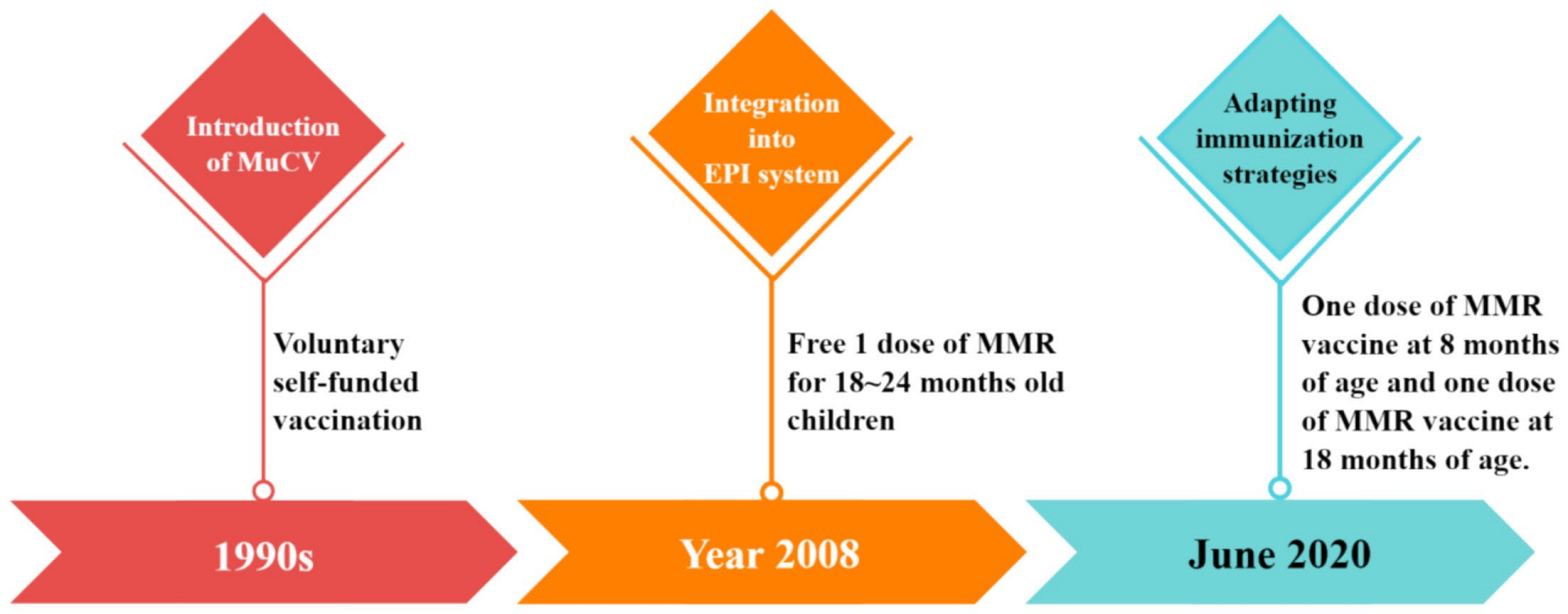
Timeline of MuCV vaccine policy development in China.

The average annual reported incidence of mumps in China from 2005 to 2023 was 18.53 /100,000 people. Mumps-containing vaccine (MuCV) was introduced in China in the 1990s, and the school-age population was voluntarily vaccinated at their own expense. In 2008, MuCV vaccination was formally incorporated into the Expanded Program on Immunization (EPI) system, and one dose of MMR vaccine was administered free of charge to children between 18 and 24 months of age, and the national immunization strategy was adjusted accordingly from June 2020, stipulating that children at 8 months and 18 months of age should be vaccinated with one dose of MMR vaccine (8) (Figure 1). A 2010–2019 surveillance study in Jiangxi Province, China, documented 90,229 mumps cases, with 91.16% (82,256 cases) occurring in children and adolescents (≤15 years) (9). In Quzhou (2006–2020), China, the vaccine effectiveness (VE) of single-dose MuCV ranged from 47.4 to 86.0%, whereas two-dose regimens achieved 64.0–92.4% VE. Although protection waned over time for both regimens, two-dose MuCV consistently provided higher protection than single-dose vaccination (10).

Since China adopted the two-dose MuCV regimen, research gaps persist in regarding mumps risk factors and VE under real-world conditions. Previous national and international studies have focused on cross-sectional investigations of mumps epidemiological trends and vaccine protection in the context of single-dose MuCV vaccination. Given the retrospective nature of this study, the use of a case-control study method is of great scientific importance. Using a 1:1 paired case-control study, this study aimed to investigate the main risk factors for mumps incidence in children under 15 years of age in Taizhou City in the context of the change from a single-dose to a two-dose MuCV vaccination strategy, to compare the differences in risk factors during the period of the different vaccination strategies, and to assess the impact of the change in vaccination strategy on the incidence of mumps. Meanwhile, secondary objectives include analyzing the association of mumps incidence with vaccination history, demographic characteristics and environmental exposures to provide data support and theoretical guidance for further improvement of mumps prevention and control strategies.

## 2 Materials and methods

### 2.1 Study design

The main objective of this paired case-control study was to investigate the main risk factors for the development of mumps in children under 15 years of age in the context of the change from a single to a two-dose MuCV vaccination strategy. Cases were selected by census. Inclusion criteria for the case group included (1) All cases of mumps with onset dates of 2021–2023 reported in the Chinese Information System for Disease Control and Prevention; (2) clinically diagnosed or laboratory-confirmed diagnosis of mumps; (3) residence in Taizhou City during the period of disease onset; and (4) age 0–14 years.

**Figure 2:**
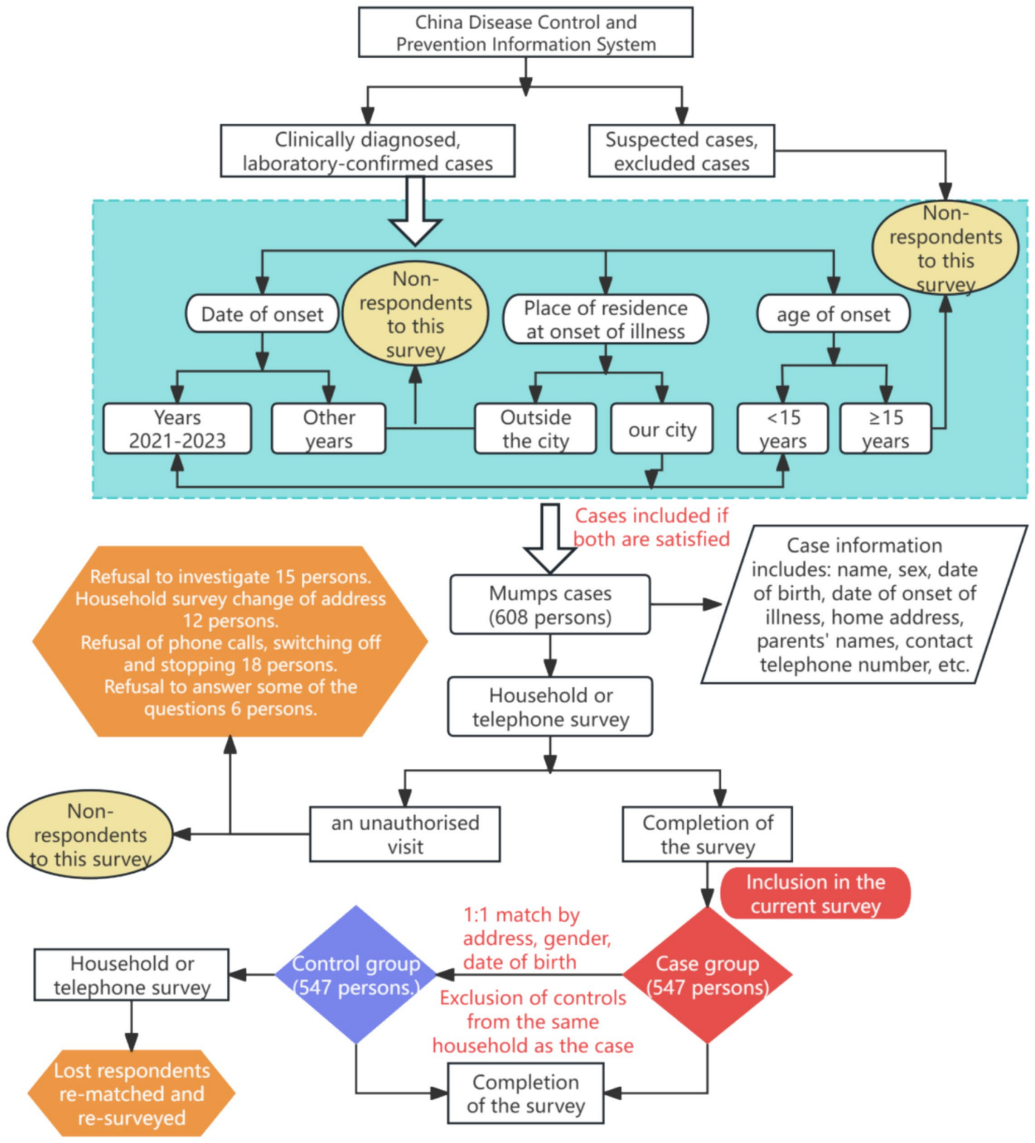
Inclusion steps for the case and control groups.

The control group was paired with the case group on a 1:1 basis, and the inclusion criteria for the control group were (1) living in the same neighborhood/street/village as the case; (2) same sex as the case; (3) same date of birth or within 30 days (±30 days) of the case; (4) Healthy children without a clinical diagnosis of mumps and with no parotid or other salivary gland symptoms as self-reported by their parents within 28 days after the matched case was fully cured (the longest incubation period for mumps is 28 days (11)); (5) not belonging to the same family as the case group (Figure 2).

For controls selected via telephone interviews, we confirmed the absence of mumps infection by verifying that: (1) No clinical or laboratory diagnosis of mumps was recorded for the child in the China CDC Information System, and (2) parents/caregivers explicitly denied any history of mumps infection in the child.

The survey was conducted from March to August 2024.

### 2.2 Sample size determination

In this study, the sample size was calculated using the events per variable (EPV) method (12, 13). With 22 independent variables encompassing socio-demographic characteristics, MuCV vaccination status, health service utilization, and awareness of related knowledge, and using “whether or not diagnosed with mumps” as the dependent variable, we applied the EPV = 15 criterion. This yielded a minimum required sample size of 330 cases (15 × 22).

In order to control the effect of potential loss to follow-up, we increased the sample size by 20%, resulting in a target sample size of at least 396 cases. Considering the mumps surveillance data from the China CDC Information System, there were a total of 608 clinically confirmed or laboratory-confirmed cases of mumps in children aged 0–14 years in Taizhou City during 2021–2023. This available case number exceeded our calculated minimum sample size (396 cases), thereby satisfying the EPV-based power requirements for multivariate analysis.

### 2.3 Content of the survey

This study employed a novel, purpose-designed questionnaire for data collection. The questionnaire was administered electronically using *Questionnaire Star*, an cost-effective online survey platform. It enabled quick creation, distribution and retrieval of questionnaires. The electronic collection replaced paper-based methods, significantly improving efficiency. It supported multiple question types and logic branching/skip patterns to flexibly meet complex survey needs and reduce transcription errors or omissions in data entry. Its data validation features like mandatory questions and format restrictions further minimized data errors from irregularities.

The survey mainly included 7 variables of socio-demographic information, 3 variables of MuCV vaccination, and 12 variables of health services and related knowledge. In addition, the exposure history and morbidity visits of the case group were examined (Table 1).

**Table 1:** Categorization and description of study variables.

Category	Variable type	Variables
Socio-demographic	Demographic	Gender
		Duration of local residence
		Birth order
		Childcare mode
	Socioeconomic	Father’s educational level
		Mother’s educational level
		Annual household per capita income
MuCV Vaccination	Vaccination	Vaccination status (yes/no)
		Number of vaccine doses
		Interval between vaccination and onset
Health services & knowledge	Behavioral	Primary healthcare facility level
		Mask-wearing at hospitals
		Mask-wearing in crowded places
		Handwashing frequency
	Knowledge	Health knowledge access
		Awareness of mumps contagiousness
	Behavioral	Self-isolation when exposed
	Attitudinal	Perceived susceptibility to mumps
		Perceived disease severity
	Behavioral	Healthcare-seeking intention if infected
	Knowledge	Knowledge: MuCV prevents mumps
	Attitudinal	Parental vaccination attitudes
Case-Specific	Epidemiological	Exposure history (case group only)
	Clinical	Morbidity visits (case group only)

**Figure 3:**
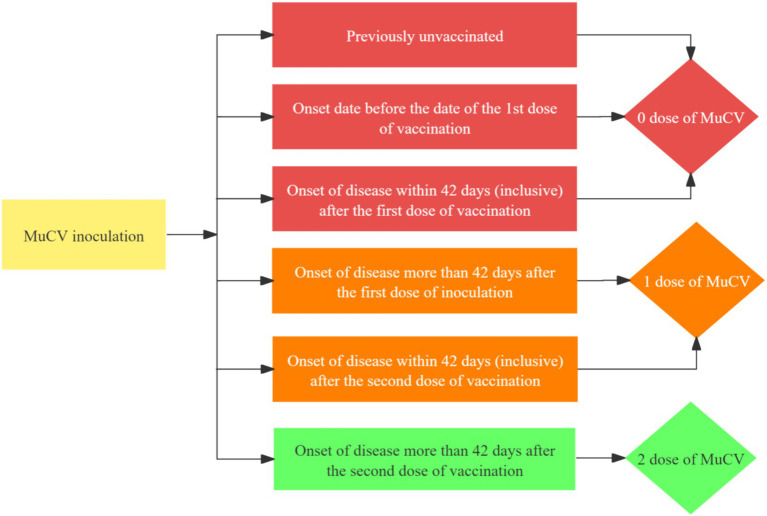
Flowchart of the method for determining the number of doses of MuCV vaccination.

MuCV vaccination information was obtainable from the Jiangsu Provincial Preventive Vaccination Comprehensive Service Management Information System or the vaccination certificate, including whether or not the vaccination was granted, the number of doses, the name of the vaccine, and the date of vaccination. Mumps breakthrough cases were mumps cases 42 days after MuCV vaccination (10). In this study, Mucv inoculation doses were determined as follows (Figure 3):

### 2.4 Quality control

The Taizhou Center for Disease Control and Prevention, as the lead unit of this project, provided technical guidance throughout and screened the study subjects in strict accordance with the established inclusion and exclusion criteria. Before the launch of the survey, experts in related fields were organized to hold a survey training meeting to optimize the research protocol, and in-depth discussions and corrections were made in response to ambiguous questionnaire items, poorly designed response options, and illogical skip patterns, among other issues.

Prior to the start of the formal survey, a pre-survey was conducted with the parents of 30 children, and the questionnaire was adjusted accordingly to address the issues set out in the questionnaire. Systematic training was also offered to professionals from the CDC at the county level. Investigators checked the content of the case questionnaire for any missing items or omissions and signed off upon completion at the end of the survey. Quality control personnel examined the questionnaire, and if problems were found, the investigator made corrections by follow-up surveys or supplemental data collection.

### 2.5 Statistical analyses

The study data were exported to Excel and analyzed using SPSS 25.0 software. Count data were presented as number of cases and percentage. Univariate and multivariate analyses were performed using conditional logistic regression analysis, with a *p*-value of <0.05 (two-tailed) as the criterion for determining statistically significant differences.

We employed conditional logistic regression analysis to identify key risk factors for mumps infection in children under 15 years old. Due to SPSS software limitations in directly performing conditional logistic regression, we implemented an established methodological alternative by adapting Cox regression models for this analysis. This approach maintains analytical validity while overcoming software constraints (12).

MuCV protective effect (VE):


$$\begin{array}{l} \mathrm{VE}=\frac{\begin{array}{l} \text{Incidence in unvaccinated population}-\\ \text{Morbidity in the vaccinated population} \end{array}}{\text{Incidence in unvaccinated population}}\;\\ \times \;100\%\;=\;{\left(1-\mathrm{RR}\right)}^{*}\;100\%\;\approx \;{\left(1-\text{OR}\right)}^{*}\;100\% \end{array}$$

(In cases of relatively low incidence, the OR is approximately equal to the RR).

### 2.6 Ethical review and informed consent

The study was formally reviewed and approved by the Ethics Review Committee of Taizhou City Hospital of Integrative Medicine (approval number: 2023-Ethics Review LW-001, approval date: 22 May 2023). All research activities were conducted in strict compliance with ethical requirements to ensure the informed consent of participants. For all children aged 0–14 years, written informed consent was obtained from their parents or guardians. This included a detailed description of the purpose of the study, the procedure, and the extent of data use. Strict confidentiality measures were also implemented throughout the study, ensuring that all data was stored and processed anonymously for research purposes only, and could not be shared with third parties without permission. The research team was committed to protecting participants’ privacy and data security.

## 3 Results

### 3.1 Descriptive characteristics

A total of 608 cases met all the above criteria, and all cases were interviewed by household or telephone survey after informed consent. Among these, 547 cases (343 males, 204 females) aged <15 years completed the survey and were included in the final analysis. The remaining 61 cases were excluded due to either uncontactable families or parental refusal. Cases were stratified into 7 groups by onset age: ≤24 months (*n* = 14), 25–48 months (*n* = 113), 49–72 months (*n* = 156), 73–96 months (*n* = 122), 97–120 months (*n* = 87), 121–144 months (*n* = 46), and ≥145 months (*n* = 9). The median age was 72 months, with children aged 49–72 months (4–6 years) representing the highest proportion of cases (28.52%, *n* = 156). Geographically, participants were distributed across 6 Taizhou districts, primarily Taixing City (38.76%) and Jiangyan District (17.92%) (Table 2).

**Table 2:** Demographic and geographical characteristics of 547 mumps case-control pairs in Taizhou City.

Features	Classification/Grouping	Number of pairs (%)
Sex	Male	343 (62.17%)
	Female	204 (37.29%)
Months of age	≤ 24	14 (2.56%)
	25–48	113 (20.66%)
	49–72	156 (28.52%)
	73–96	122 (22.30%)
	97–120	87 (15.90%)
	121–144	46 (8.41%)
	≥ 145	9 (1.65%)
Distribution of current addresses	Hailing district	97 (17.73%)
	Pharmaceutical Hi-Tech Zone (Gaogang District)	44 (8.04%)
	Jiangyan district	98 (17.92%)
	Xinghua district	33 (6.03%)
	Jingjiang district	63 (11.52%)
	Taixing district	212 (38.76%)

### 3.2 Univariate analysis of sociodemographic characteristics of survey respondents

Seven variables of demographic characteristics such as gender, duration of local residence (length of residence in a given area), number of births, mode of care, educational level of father/mother, and annual per capita household income were included in conditional logistic regression for univariate analyses for each of the seven variables, with case group = 1 and control group = 0. The results showed that duration of local residence was a protective factor for the incidence of the mumps, and that the longer the local time of residence was, the less susceptible to contracting the mumps (OR = 0.662, 95% CI 0.519 ~ 0.844). The number of births and the type of care were risk factors for the development of mumps, the more births, the more likely to be infected with mumps (OR = 1.488, 95% CI 1.157 ~ 1.913), and children cared for by other people were more likely to be infected with mumps compared to those cared for by their parents (OR = 2.417, 95% CI 1.502 ~ 3.889). There was no statistically significant difference between educational level of father/mother, annual per capita household income and the incidence of mumps (*p*-value>0.05) (Table 3).

**Table 3:** Univariate analysis of different socio-demographic characteristics and risk factors for the development of mumps.

Variable	Control group	Case group	*B-*value	Wald *χ*^2^ value	*p-*value	Exp (B)	95% CI	
	Numbers (Composition ratio)	Numbers (Composition ratio)					Lower limit	Upper limit
Time of residence in the local area			−0.413	11.11	<0.01^*^	0.662	0.519	0.844
<3 months	1 (0.2%)	7 (1.3%)						
3 months - 1 year	6 (1.1%)	12 (2.2%)						
1–5 years	155 (28.3%)	183 (33.5%)						
Over 5 years	385 (70.4%)	345 (63.1%)						
Number of births			0.397	9.602	<0.01^*^	1.488	1.157	1.913
1 Tire	400 (73.1%)	348 (63.6%)						
2 children	141 (25.8%)	195 (35.6%)						
3 and above	6 (1.1%)	4 (0.7%)						
Care mode			0.882	13.217	<0.01^*^	2.417	1.502	3.889
Parent-led	512 (93.6%)	478 (87.4%)						
Other	35 (6.4%)	69 (12.6%)						
Father’s education level			−0.142	2.246	0.134	0.868	0.721	1.045
Junior high school and below	56 (10.2%)	83 (15.2%)						
High school/Junior college	187 (34.2%)	165 (30.2%)						
University and above	304 (55.6%)	299 (54.7%)						
Mother’s education level			−0.048	0.262	0.609	0.953	0.794	1.144
Junior high school and below	72 (13.2%)	97 (17.7%)						
High school/Junior college	192 (35.1%)	153 (28.0%)						
University and above	283 (51.7%)	297 (54.3%)						
Annual per capita household income			0.147	1.388	0.239	1.159	0.907	1.481
Less than 20,000	21 (3.8%)	26 (4.8%)						
20,000-40,000	114 (20.8%)	85 (15.5%)						
More than 40,000	412 (75.3%)	436 (79.7%)						

### 3.3 Univariate analysis of MuCV vaccination among survey respondents

Three variables, MuCV vaccination history, number of doses and post-vaccination interval, were included in conditional logistic regression for univariate analysis, and the results showed that Vaccination history was a protective factor for the development of mumps, and vaccination with MuCV was associated with a lower likelihood of developing mumps (OR = 0.143, 95% CI 0.056 ~ 0.365) and a VE of 85.7% (63.5% ~ 94.4%). The number of vaccination doses was a protective factor for the development of mumps, with more vaccination doses being less likely to be infected with mumps (OR = 0.312, 95% CI 0.182 ~ 0.504) and VE was 68.8% (46.6% ~ 81.8%). Subgroup analysis being a function of the interval between the most recent MuCV vaccination and the time of case onset showed no statistically significant difference (*p*-value>0.05) (Table 4).

**Table 4:** Univariate analysis of MuCV vaccination-related information and risk factors for mumps development.

Variable	Control group	Case group	*B-*value	Wald *χ*2 value	*p-*value	Exp (B) (95% CI)	VE (%) (95% CI)
	Numbers (Composition ratio)	Numbers (Composition ratio)					
Vaccination history			−1.946	16.566	<0.01^*^	0.143 (0.056 ~ 0.365)	85.7 (63.5 ~ 94.4)
No	7 (1.3%)	37 (6.8%)					
Yes	540 (98.7%)	510 (93.2%)					
Number of doses			−1.165	18.092	0.000	0.312 (0.182 ~ 0.534)	68.8 (46.6 ~ 81.8)
0 doses	7 (1.3%)	37 (6.8%)					
1 doses	471 (86.1%)	451 (82.4%)					
2 doses	69 (12.6%)	59 (10.8%)					
Time between the last MuCV vaccination and the onset of the disease			0.136	1.143	0.285		
<5 years	315 (57.6%)	316 (57.8%)					
5 ~ 10 years	202 (36.9%)	191 (34.9%)					
10 ~ 15 years	12 (2.2%)	14 (2.6%)					
Unvaccinated	18 (3.3%)	26 (4.8%)					

### 3.4 Univariate analysis of respondents’ health services and related knowledge

Health services and related knowledge were included in separate conditional logistic regressions for univariate analyses of factors showed that there was no statistically significant difference between whether or not they washed their hands frequently, had different access to health knowledge, whether or not they would actively isolate themselves from others in their neighborhood when they were infected with the mumps, and whether or not they would choose to go to the hospital and the onset of the mumps (*p*-value>0.05). The level of the most frequently visited hospital (OR = 1.962, 95% CI 1.551 ~ 2.482), whether masks were worn when going to a hospital (OR = 1.770, 95% CI 1.293 ~ 2.423) or a crowded indoor place (OR = 1.781, 95% CI 1.387 ~ 2.287), whether mumps is a contagious disease (OR = 1.940, 95% CI 1.491 ~ 2.525), awareness of the severity of mumps (OR = 1.392, 95% CI 1.206 ~ 1.607), whether MuCV vaccination can prevent mumps (OR = 1.610, 95% CI 1.222 ~ 2.121), whether parents had a positive attitude toward vaccination (OR = 1.447, 95% CI 1.112 ~ 1.882) were risk factors for developing mumps (all *p*-value<0.01). Whether the children were infected with mumps in the future (OR = 0.280, 95% CI 0.190–0.411) was considered a protective factor for the development of mumps (*p*-value <0.01) (Table 5).

**Table 5:** Univariate analysis of health services and related knowledge and risk factors for mumps incidence.

Variable	Control group	Case group	*B-*value	Wald *χ*2 value	*p-*value	Exp (B)	95% CI	
	Numbers (Composition ratio)	Numbers (Composition ratio)					lower limit	upper limit
The level of the most frequently visited hospital			0.674	31.556	<0.01^*^	1.962	1.551	2.482
Township hospital and below	134 (24.5%)	60 (11.0%)						
County level	257 (47.0%)	294 (53.7%)						
Municipal and above	156 (28.5%)	193 (35.3%)						
Whether masks were worn when going to a hospital			0.571	12.721	<0.01^*^	1.770	1.293	2.423
Yes	125 (22.9%)	78 (14.3%)						
No	422 (77.2%)	469 (85.7%)						
Whether masks were worn when going to a crowded indoor place			0.577	20.492	<0.01^*^	1.781	1.387	2.287
Yes	275 (50.3%)	200 (36.6%)						
No	272 (49.7%)	347 (63.4%)						
Do you wash your hands frequently			0.439	3.404	0.065	1.552	0.973	2.475
Yes	514 (94.0%)	498 (91.0%)						
No	33 (6.0%)	49 (9.0%)						
Access to health information			0.091	1.455	0.228	1.096	0.944	1.271
Relatives/friends/teachers/medical personnel	177 (32.4%)	157 (28.7%)						
Radio and television/newspapers and periodicals	39 (7.1%)	47 (8.6%)						
Internet	331 (60.5%)	343 (62.7%)						
Whether mumps is a contagious disease			0.663	24.362	<0.01^*^	1.940	1.491	2.525
Yes	251 (45.9%)	172 (31.4%)						
No	296 (54.1%)	375 (68.6%)						
Do you take the initiative to isolate people around you when they are infected			−0.120	0.477	0.490	0.887	0.632	1.246
Yes	455 (83.2%)	463 (84.6%)						
No	92 (16.8%)	84 (15.4%)						
Whether the children were infected with mumps in the future			−1.274	41.868	<0.01^*^	0.280	0.190	0.411
Yes	55 (10.1%)	140 (25.6%)						
No	492 (89.9%)	407 (74.4%)						
Awareness of the severity of mumps			0.331	20.443	<0.01^*^	1.392	1.206	1.607
Severe	220 (40.2%)	218 (39.9%)						
Fairly serious	191 (34.9%)	64 (11.7%)						
Not serious	136 (24.9%)	265 (48.4%)						
If a child is infected with mumps, will he/she choose to seek medical treatment			−0.693	3.203	0.074	0.500	0.234	1.068
Yes	527 (96.3%)	537 (98.2%)						
No	20 (3.7%)	10 (1.8%)						
Whether MuCV can prevent mumps			0.476	11.464	<0.01^*^	1.610	1.222	2.121
Yes	287 (52.5%)	237 (43.3%)						
No	260 (47.5%)	310 (56.7%)						
Whether parents had a positive attitude toward vaccination			0.369	7.583	<0.01^*^	1.447	1.112	1.882
Yes	392 (71.7%)	350 (64.0%)						
No	155 (28.3%)	197 (36.0%)						

### 3.5 Multifactorial analysis of factors affecting the incidence of mumps

The case and control groups were invoked as dependent variables, where the case group was assigned a value of 1 and the control group was assigned a value of 0. Multifactor conditional logistic regression included variables that were statistically significant in the univariate analysis, and further analyses showed that: Longer local residence (OR = 0.548, 95% CI 0.403 ~ 0.744), history of MuCV vaccination (OR = 0.103, 95% CI 0.036 ~ 0.297), and belief that children would not get mumps (OR = 0.197, 95% CI 0.121 ~ 0.319) reduced the risk of mumps infection. However, families with multiple births (OR = 1.926, 95% CI 1.405 ~ 2.640), being cared by someone other than parents (e.g., grandparents, babysitters, relatives or staff at a childcare center) (OR = 4.366, 95% CI 2.417 ~ 7.888), a higher level of the most frequently visited hospital (OR = 2.012, 95% CI 1.490 ~ 2.716), going into a crowded indoor place without wearing a mask (OR = 1.699, 95% CI 1.237 ~ 2.334), believing that mumps is not an infectious disease (OR = 1.782, 95% CI 1.274 ~ 2.492), believing that the disease is not serious (OR = 1.507, 95% CI 1.260 ~ 1.802), and believing that MuCV cannot prevent mumps (OR = 2.052, 95% CI 1.451 ~ 2.901) increased the risk of mumps infection (Table 6).

**Table 6:** Multivariate analysis of risk factors for the incidence of mumps in children under 15 years of age.

Variant	*B-*value	Wald *χ*^2^ value	*p-*value	Exp (B)	95% CI	
					lower limit	upper limit
Local time of residence	−0.602	14.868	<0.01^*^	0.548	0.403	0.744
Number of births	0.655	16.600	<0.01^*^	1.926	1.405	2.640
Type of care	1.474	23.851	<0.01^*^	4.366	2.417	7.888
MuCV vaccination history	−2.270	17.802	<0.01^*^	0.103	0.036	0.297
Level of hospital most frequently visited	0.699	20.827	<0.01^*^	2.012	1.490	2.716
Wear a mask when going to densely populated indoor places	0.530	10.705	<0.01^*^	1.699	1.237	2.334
Is mumps a contagious disease	0.578	11.394	<0.01^*^	1.782	1.274	2.492
Whether the child will be infected with mumps in the future	−1.625	43.370	<0.01^*^	0.197	0.121	0.319
Perceived severity of mumps	0.410	20.209	<0.01^*^	1.507	1.260	1.802
MuCV can prevent mumps	0.719	16.537	<0.01^*^	2.052	1.451	2.901

### 3.6 Case exposure history and morbidity visits

The case group was surveyed regarding exposure history and morbidity visits, and the results showed that: in the 21 days before the symptoms of parotid swelling appeared, the proportion of children who went to non-medical institutions and densely populated places was quite high, amounting to 44.61%, while the proportion of those who went to foreign places was the lowest, amounting to only 2.74%; 91.41% of the parents took the protective measure of wearing masks for their children when taking them to the health care institutions. After the diagnosis of mumps was confirmed by doctors, 88.48% of them gave home quarantine advice, 68.74% advised to reduce contact with other children, 29.98% advocated avoiding public transport, and 53.75% stressed that the windows of the residence should be kept open for ventilation; In addition, 8.41 per cent recommended MuCV for those who had contact with cases and lacked a history of immunization (Table 7).

**Table 7:** Exposure history and morbidity visits for mumps cases in children under 15 years of age in Taizhou City.

Variant		Numbers	Component ratio (%)
Within 21 days prior to parotid swelling	Visited hospitals/clinics/vaccination units	90	16.45
	Visited other densely populated places	244	44.61
	Been out of town	15	2.74
	Someone has come (returned) to your home from out of town	17	3.11
	Has been in contact with someone who has a fever and swollen parotid glands	82	14.99
When taking a child to a hospital	Masking children	500	91.41
After the diagnosis of runny cheeks	Exposure to children <15 years of age	147	26.87
When the doctor diagnosed a runny cheek, he told	Home isolation is recommended	484	88.48
	Less contact with other children	376	68.74
	Avoid public transportation	164	29.98
	Opening of windows and ventilation of the residence	294	53.75
	Persons in contact with cases with no history of immunization to be vaccinated with MuCV	46	8.41

## 4 Discussion

Since the adjustment of our MMR immunization strategy in 2020, there has been no relevant study on the risk factors for the development of mumps, and this study addresses this gap. In this study, conditional logistic regression analysis was used to investigate the main risk factors for the development of mumps in children under 15 years of age.

The study have shown that children who have lived at their current address for <3 months are at higher risk of mumps, a finding that is consistent with the results of an Australian study (14) and with the epidemiological characteristics of other respiratory infections (15–17). In the current society, population mobility has increased significantly, which poses numerous challenges in public health, and the continuous migration of population dramatically increases the transmission rate of infectious diseases (18). VE is a measure of the evaluation of the protective effect of vaccines on specific populations, and domestic and international studies have shown that the VE of MuCV is 75 to 95% (19), and in a study in Yancheng City, Jiangsu Province, the MuCV VE was 80.38% (20). Univariate logistic regression analysis showed that receiving two doses of MuCV vaccine provided better protection compared to one or zero doses, a result consistent with the findings of previous studies (10, 21–23).

The study showed that there was no statistical difference between the time of the most recent MuCV vaccination and the time interval between the onset of cases and the onset of mumps in university analyses, which was different from the trend of decreasing vaccine protective efficacy over time in previous studies (24–26), suggesting that there is a need for follow-up studies to monitor the antibody levels of the population at different intervals of time after MuCV vaccination and to study the attenuation of the vaccine protective efficacy over time in a Patterns. There was not any statistically significant difference between the number of MuCV vaccination doses and the incidence of mumps in the multifactorial analysis, which was inconsistent with the results of the studies in Shandong province and Liuzhou city (11, 27). Taizhou city has been implementing a two-dose MMR immunization strategy since June 2020, and due to the short observation period, there were relatively fewer two-dose vaccine recipients among children with onset of disease in 2021–2023, and the time intervals between them and the time since the vaccine, resulting in a Uneven distribution of the composition ratio of vaccination doses, which may result in a statistically significant difference between the number of vaccination doses and the onset of MMR not being detected.

The study have demonstrated that MuCV vaccination is a protective factor for the development of mumps. Among WHO member countries, 63.40% have found at least one MuCV vaccination in their national immunization programs (28). The success of those countries and territories that have reached the goal of controlling or eliminating mumps has been the maintenance of high levels of MuCV vaccination rates through routine immunization programs (29). The reasons for believing that children will not be infected with mumps in the future and that it is a protective factor for the development of mumps may be manifold; firstly, it may be because these children have already been vaccinated with MuCV and their parents have a high-level of confidence in the protective effect of the vaccine. Secondly, these parents may be highly conscious of the protective measures for their children in their daily lives, in addition to the parents’ belief that the children have already been infected with mumps and have boosted immunity in their bodies and will not be re-infected.

The study have shown that multiple birth families and non-parental care patterns are significant risk factors for the development of mumps. In particular, children in large families are at significantly increased risk of viral transmission due to prolonged exposure to shared living, learning and recreational environments. Notably, this finding differs from the epidemiological profile of respiratory infections. For example, studies of risk factors for influenza incidence have found no statistically significant association between the number of children in the household and influenza incidence (30). Studies have shown that when children are cared for by non-parental caregivers (including grandparents, babysitters, relatives, or childcare center staff), they are at significantly increased risk of exposure to mumps due to several factors: first, non-parental caregivers may have inadequate knowledge of the child’s health status and vaccination history; second, they may have difficulty recognizing and assessing the risk of mumps exposure in a timely manner; and finally, there may be a delay in risk identification and implementation of protective measures. This finding is consistent with previous findings (31) that children in childcare settings are at higher risk of respiratory infections, which may be related to increased opportunities for intimate contact and cross-infection in group living environments. The most frequent visit to high level hospitals is a risk factor for mumps incidence, which is consistent with the results of the factors influencing the incidence of measles in Changzhou (32), which may be due to the following four reasons: firstly, high level hospitals are usually located in large cities, with high flow of people and high patient density, so there are more chances of contacting patients with mumps, and the risk of viral transmission is higher, especially in the diagnostic and treatment process, which may have more direct contact with infected persons, thus increasing the risk of cross-infection. In particular, there may be more direct contact with infected persons during consultation and treatment, increasing the likelihood of cross-infection. Secondly, patients attending high level healthcare facilities may include people with relatively weakened immunity, who may not have followed the MuCV vaccination program and thus lie at higher risk of mumps virus infection. Thirdly, children who frequently travel to high level hospitals may be low and vulnerable to communicable diseases. Fourthly, healthcare workers are possible vectors of mumps transmission, especially those who have not meant vaccinated with MuCV or have not meant fully vaccinated.

The study have shown that traveling to densely populated indoor places without wearing a mask is a risk factor for the onset of mumps, and previous studies have shown that wearing a mask is effective in interrupting the transmission of respiratory infections such as mumps (11, 33), probably because in dense indoor places with poor air circulation, there tends to be closer contact with others in a short period of time, and the absence of a mask increases the risk of contact with infected people, making the virus easier to spread in groups. Some parents incorrectly believe that mumps does not constitute an infective disease, that the condition is not serious, and that MuCV is not helpful in preventing mumps, all of which are risk factors for the development of mumps. These parents have obvious deficiencies in their knowledge of mumps, underestimate the strength of the contagiousness of the infection and the potentially serious risks, which leads to a weak sense of precautionary awareness of mumps, failure to take timely and effective precautionary measures, as well as failure to bring their children to seek medical treatment in a timely manner. In addition, due to misconceptions about the effectiveness of the vaccine, some parents may opt not to have their children vaccinated with MuCV, thereby increasing the likelihood of infection and complications.

For effective mumps prevention and control, a multi-pronged strategy is recommended. Coordinated efforts among government agencies, CDCs, healthcare facilities, and schools should jointly implement digitally supported health education programs, emphasizing key transmission routes (primarily respiratory droplets, secondarily fomite contact), cardinal symptoms (parotitis/fever), and vaccination benefits. Vaccination efforts should prioritize verifiable high-risk groups, including children aged 4–6 years, migrant children (via cross-regional immunization records), under-vaccinated children (tracked through immunization information systems), and daycare attendees/multi-child households, supported by SMS-based reminder systems. Enhanced surveillance should incorporate RT-PCR testing for suspected cases (fever with parotid swelling), 5-day isolation protocols, and focused monitoring of transient populations (<3-month residency) and children in non-parental care arrangements. Concurrently, family-centered prevention should reinforce hand hygiene, surface disinfection, and caregiver education on warning signs, ensuring priority protection for epidemiologically vulnerable subgroups.

## 5 Conclusion

This study analyzed the risk factors for mumps incidence in children under 15 years of age in the context of the switch from a single to a two-dose MMR vaccination strategy and found that timely vaccination with two doses of MuCV vaccine was a key protective factor against mumps, whereas factors such as missed vaccinations, short local residence, multiparous families, non-parental care and frequent travel to densely populated areas without wearing a face mask significantly increased the risk of mumps incidence. The results of the study provide an important scientific basis for the development and optimisation of mumps prevention and control strategies.

The study supports that further promotion of two doses of MuCV vaccine in existing immunization programs, especially among migrant children and children with delayed vaccination, can significantly reduce the incidence of mumps. Timely provision of two doses of MuCV vaccine is a key component of the prevention and control strategy, as two doses of vaccine can radically improve immunoprotection and effectively interrupt the chain of transmission of mumps. However, vigilance must be maintained against possible breakthrough infections. Although vaccination can substantially reduce morbidity, breakthrough cases can still occur if vaccination coverage is inadequate or if the virus mutates. Therefore, continuous monitoring and evaluation of vaccine efficacy and timely adjustment of prevention and control strategies are important safeguards to ensure the long-term effectiveness of vaccination programs.

Accurate identification of high-risk areas and high-risk groups (e.g., short-stay families, multiple births, etc.) will provide a scientific basis for optimal allocation of mumps prevention and control resources and targeted interventions, thereby increasing the efficiency of prevention and control. Meanwhile, raising public awareness of mumps and its vaccine and correcting misconceptions such as “mumps is not an infectious disease” or “MuCV cannot prevent mumps” will help increase vaccination rates and reduce vaccine hesitancy. In addition, the study suggests that the active surveillance system for mumps cases should be further improved to reduce under reporting and misreporting and to provide more reliable data for mumps prevention and control.

### 5.1 Strengths and limitations

The strength of this study is that it focuses on the risk factors for mumps incidence in the context of the switch from a single-dose to a two-dose MMR vaccination strategy and uses a 1:1 paired case-control study design, which effectively controls for confounding factors and improves the reliability of the study results. Case data were obtained from the China Information System for Disease Control and Prevention, which ensured the authority and representativeness of the data. By analyzing various risk factors such as vaccination history, demographic characteristics, environmental exposures, etc., the factors influencing the incidence of mumps were comprehensively revealed, which provided an important reference for the development and adjustment of mumps prevention and control strategies and was of great public health significance.

However, there are some limitations to this study. First, the data on mumps cases were obtained from a passive surveillance reporting system, which may be subject to omission and misreporting, and the study relied on retrospective data and parent-reported information from questionnaires, which may be subject to recall bias; second, although a matched-pair design was employed, residual confounding (e.g., unmeasured household hygiene practices or undocumented close-contact exposures) may have influenced the results, and potential misclassification of MuCV immunization status (partial vs. full immunity) could occur due to reliance on recall or incomplete records. The study likely underrepresented true mumps incidence, as subclinical or mild cases not seeking medical attention were excluded from case ascertainment; furthermore, the study only included data from 2021 to 2023, a relatively short time period that may not fully reflect the impact of adjustments in long-term immunization strategies.

There were further limitations in sample selection. The initial case group included 608 cases, but only 547 were successfully contacted, representing a loss to follow-up of 10%. This loss to follow-up may have introduced selection bias, as households with higher vaccine awareness or greater willingness to engage with the public health system may have been more likely to take part in the survey. However, even in the case group, we observed resistance to vaccines and the public health system among some households, suggesting that the loss of visits may not be entirely due to positive attitudes, but rather reflects alienation or distrust of the public health system among some segments of the population. This skewed loss of visits challenges the interpretation of the study results and highlights a key issue in public health policy making: how to engage those who are antagonistic or alienated from the public health system in immunization planning and other health interventions. This is a major problem, but also a necessary condition for achieving universal health coverage. Future research should continue to explore ways to increase the trust and participation of these populations in the public health system through community engagement, health education and other strategies to ensure the effectiveness and equity of immunization planning.

With regard to the applicability of the study results, differences in vaccination schedules in different provinces or countries, the potential impact of differences in mumps virus genotypes prevalent in different places on vaccine efficacy, and differences in the implementation of the newly introduced MMR vaccination program in 2020 in different places, which include vaccination coverage, vaccination schedules, vaccine availability and the degree of sophistication of the surveillance system, among other factors, must be taken into account. In addition, the retrospective design and timeframe of this study may not fully capture potential cyclical outbreak patterns beyond 2023, a nuance that suggests the need to consider the results of the study in a longer-term temporal context when interpreting them. These limitations suggest that caution should be exercised in interpreting the results and also point the way to future research, which should include metacentre studies to cover different regions and time periods, strengthening active surveillance systems to improve completeness of case reporting, considering the inclusion of viral genotypic to assess its impact on vaccine efficacy, and conducting long-term follow-up studies to assess the long-term effects of the vaccination strategy, in order to have a more complete understanding of the effects of MuCV and to provide more reliable evidence for optimizing vaccination strategies.

## Data Availability

The raw data supporting the conclusions of this article will be made available by the authors, without undue reservation.
