# Supplementary material for: Risk factors for mumps in children under 15 years of age during the transition from single-dose to two-dose MMR vaccination strategy: a matched case-control study
Source: Front Public Health. 2025 Jul 7;13:1592602. doi: 10.3389/fpubh.2025.1592602 (PMC12277324; doi:10.3389/fpubh.2025.1592602)
Supplement: Supplementary file 1 [file Data_Sheet_1.docx]

Paired case-control survey of mumps transmission factors among people under 15 years of age in Taizhou City, China

Case Name：

Control Name：

（For controls, please fill in both the case name and control name）

Investigator Signature：

Investigator Affiliation：

Investigator Phone：

Date of Investigation: Year Month Day

Before the investigation, please inform the participants that the collected information will be kept strictly confidential, and there are no right or wrong answers—truthful responses are essential.

Taizhou Center for Disease Control and Prevention

| Part I Basic Information | | | | | | | Options |
| --- | --- | --- | --- | --- | --- | --- | --- |
| 1.Sex | | | | 1=male, 2=female | | |  |
| 2.Date of birth (fill in the calendar date) | | | | Year Month Day | | |  |
| 3.Present address //Street Village/Subdivision | | | | City District Township Street Village/Subdivision | | |  |
| 4.How long has your child lived at your current address | | | | 1=“<3 months” 2=“3 months-1 year” 3=“1-5 years” 4="Over 5 years “More than 5 years” | | |  |
| 5.This child of yours is the first ( ) child | | | | 1= 1st, 2= 2nd, 3= 3rd or more | | |  |
| 6.Who is your child's primary caregiver during the week (check one) | | | | 1= Parent, 2= Grandparent, 3= Nanny, 4= Other | | |  |
| a) If not parents, age of primary caregiver | | | | Years old | | |  |
| b) If not parents, why | | | | 1= both parents work outside the home, 2= father/mother is away on business for a long time, 3= parents are divorced, 4= orphaned, 5= other reasons | | |  |
| 7.Education level of the child's mother | | | | 1=Junior high school and below, 2=High school/Junior college, 3=University and above | | |  |
| 8.Education level of the child's father | | | | 1=Junior high school and below, 2=High school/Junior college, 3=University and above | | |  |
| 9.your family's per capita annual income is ( ) RMB | | | | 1=“less than 20,000”, 2=“20,000-40,000”, 3="more than 40,000” | | |  |
| 10.The respondent is the survey respondent's | | | | 1=mother, 2=father, 3=grandparent, 4=other | | |  |
| 11.respondent contact number (as far as possible for the cell phone number) | | | | Cell phone: Fixed phone: | | |  |
| Part II Mumps or MMR Vaccination History | | | | | | | Options |
| 12.Does the child have a vaccination certificate | | | | 1=Yes, 2=No, 99=Don't know | | |  |
| 13.Please transcribe all the vaccination information of the child's mumps vaccine (the investigator will determine the number of doses of mumps vaccine according to the date of birth on the spot, and fill in the information item by item and ask for the information of each dose based on the vaccination certificate when entering the household; if there is no vaccination certificate/card on the spot, the investigator will try to check with the Preventive Vaccination Information System) | | | | | | |  |
| Type of vaccination dose | Each dose of mumps vaccine or MMR vaccineDetailed information of vaccination (only fill in the dose that has been recorded exactly) | | | | If no vaccination certificate/card is available at the site, parents should try to recall whether or not they have been vaccinated.  1=Yes , 2=No, 99=Don't know | Reason for not vaccinated(In order of priority, from the most important to the least important reasons, fill in the space below from left to right, maximum 3 items) |  |
|  | Name of vaccine | Manufacturer | Date of vaccination（Format 2022-01-01） | Source of information (single choice)  (1=vaccination certificate, 2=vaccination card, 3=information system) |  |  |  |
| 1st dose |  |  |  |  |  |  |  |
| 2nd dose |  |  |  |  |  |  |  |
| Other |  |  |  |  |  |  |  |
| List of reasons for non-vaccination  A: Subjective reasons of the vaccinated party  A1: Don't think the disease is serious  A2: Don't think the vaccine is effective  A3: The child is in good health and is not prone to getting the disease  A4: Think it is more natural to get sick  A5: Don't vaccinate for fear of side effects  A6: Have been vaccinated before and don't want to be vaccinated again  A7: Previously had measles, already have resistance  A8: Religious or customary practices  B: Objective reasons of the vaccinee  B1: Adults don't have time to bring the child for vaccination  B2: Don't know the time/place for vaccination  B3: Don't have local household registration and worry about not being vaccinated  B4: Forgot to make an appointment for vaccination  B5: Was not at the vaccination place at that time  B6: The child had contraindications and could not be vaccinated  B7: Didn't know that the vaccine was to be vaccinated  C: Reasons related to the provision of vaccination services  C1: Vaccination points are set too far away or not easily accessible  C2: Failure to vaccinate due to shortage of vaccine supply  C3: Charges for vaccines  C4: Poor service at the vaccination center  C5: Failure to provide vaccination service in time  D: Other  D1：Other (specify reason ) | | | | | | |  |

| Part III Health Services and Related Knowledge | | | Options |
| --- | --- | --- | --- |
| 14.When your child is sick, the level of hospital you go to most often | 1=Provincial 2=Prefecture, 2=Municipal, 3=County 4=Street/township health center,  5=Village doctor, 6=Private clinic, 7=Never sought medical treatment, 99=Don't know | |  |
| a) Distance of the hospital from your home | kilometers | |  |
| b) Whether it is convenient to go to the hospital | 1=Yes, 2=No, 99=Don't know | 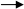If option 1, skip to  question 15 |  |
| c) Most important reason for inconvenient access to medical care (single choice) | 1=The hospital is too far away or the transportation is inconvenient, 2=Too busy at work to seek medical treatment,3=The cost of medical care is too high, 4=No local household registration, have worries about seeking medical treatment, 5=Other reasons (specify ), 99=Don't know | |  |
| 15.Wear a mask when your child goes to the hospital? | 1=Yes, 2=No, 99=Don't know | |  |
| 16.Whether the child often wears a mask when going to other indoor public places. | 1=Yes, 2=No, 99=Don't know | |  |
| 17.Whether your child pays attention to hand hygiene and washes his/her hands frequently. | 1=Yes, 2=No, 99=Don't know | |  |
| 18.What is the most important way for you to get information about health knowledge?(single choice) | 1=Relatives and friends, 2=Health care workers, 3=Radio and television, 4=Newspapers and periodicals, 5=Internet, 6=Others( ) | |  |
| 19.Do you think mumps is an infectious disease? | 1=Yes, 2=No, 99=Don't know | 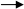If option 1, continue with question (a) below.  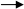If you choose 2 or 99, go to question 20. |  |
| a) If yes, how do you think mumps is spread (multiple choice) | 1=Airborne, 2=Contact, 3=Fecal-oral, 99=Don't know | |  |
| 20.When a loved one or friend has the mumps, do you have your child actively isolate themselves from that person | 1=Yes, 2=No, 99=Don't know | |  |
| 21.Do you think your child will get mumps in the future? | 1=Yes 99=Don't know  2=No | 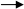Jump to question 22  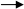If option 2, continue with question (a) below. |  |
| a) If no, the most important reason why you think you will not get mumps (multiple choice) | 1=have had mumps 2=have been vaccinated, 3=There are fewer mumps patients, so there is no chance to get infected,  4=Think you have a strong body resistance,  5=Other( ) | |  |
| 22.If your child has mumps, do you think it is serious? | 1=Yes, 2=No, 99=Don't know | |  |
| 23.If your child has mumps, do you take him to the hospital? | 1=Yes, 2=No, 99=Don't know | |  |
| 24.Do you think the mumps vaccine can prevent mumps? | 1=Yes, 2=Partially preventable, 3=No, 99=Don't know | |  |
| 25.Would you like to give your child another dose of mumps-containing vaccine if booster immunization is offered? | 1=Yes 99=Don't know  2=No | 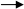If option 2, continue with question (a) below. |  |
| a) if no, why do you not want to be vaccinated (select in order of importance, up to 3 reasons)    (most importantly) (second most important) (third most important) | 1=Didn't think the disease was serious, 2=Didn't think the vaccine was effective, 3=Think it is more natural to get the disease, 4=Don't want to be vaccinated because of side effects, 5=I have been vaccinated in the past and do not want to be vaccinated again, 6=I have been vaccinated against mumps in the past and already have resistance to it, 7=Religion or custom does not accept the vaccine, 8=I am in good health, I am not prone to get it,  9=Other( ), 99=Don't know | |  |
| **The above 25 questions must be answered by both cases and controls** | | | |
| Part IV Case Exposure History and Morbidity Visits (case responses only) | | | Options |
| 26.Has your child visited a hospital/clinic/vaccination unit for any reason (e.g., doctor's appointment, visit, vaccination) in the 21 days before the parotid swelling? | 1=Yes, 2=No, 99=Don't know | |  |
| 27.Has your child been to a populated indoor place other than a hospital in the 21 days before parotid swelling? | 1=Yes, 2=No, 99=Don't know | |  |
| 28.Has your child traveled outside the country during the 21 days before parotid swelling? | 1=Yes, 2=No, 99=Don't know | |  |
| 29.During the 21 days before your child's parotid swelling, did anyone from out of town come to (or return to) your home? | 1=Yes, 2=No, 99=Don't know | |  |
| 30.Has your child been in contact with anyone with fever and swollen parotid glands in the 21 days before the swelling? | 1=Yes, 2=No, 99=Don't know | |  |
| 31.Did you put a mask on your child when you took him/her to the hospital with mumps? | 1=Yes, 2=No, 99=Don't know | |  |
| 32.Has the child been in contact with children <15 years old after diagnosis of mumps? | 1=Yes, 2=No, 99=Don't know | |  |
| 33.When the doctor diagnosed mumps, did he/she tell some precautions? (The investigator asked the respondents to give open-ended answers and choose according to the answers, multiple choices are allowed) | 1=Isolation at home is recommended, 2=Less contact with other children, 3=Avoid public transportation, 4=Open windows and ventilate your home, 5=People in contact with the case should be vaccinated against mumps if they have no history of immunization, 6=Others( ) | |  |
| *Please check that the questionnaire is completed before leaving the respondent's home. After checking, the surveyor will fill in and answer the following questions* | | | |
| ★Whether the questionnaire was completed (by the investigator) | 1=All questions were asked and filled out, 2=Some of the questions were not filled out and the respondents could not understand the questions, 3=Some questions could not be filled in, other reasons（ ） | |  |
