# Supplementary material for: Risk factors for mumps in children under 15 years of age during the transition from single-dose to two-dose MMR vaccination strategy: a matched case-control study
Source: Front Public Health. 2025 Jul 7;13:1592602. doi: 10.3389/fpubh.2025.1592602 (PMC12277324; doi:10.3389/fpubh.2025.1592602)
Supplement: Supplementary file 2 [file Data_Sheet_2.docx]

**Informed Consent Form for the Matched Case-Control Study on Transmission Factors of Mumps in Children Under 15 Years Old in Taizhou City**

Dear Parents/Guardians:

Greetings!

We are conducting a paired case-control study on the transmission factors of mumps among people under 15 years of age in Taizhou City. The purpose of this study is to understand the transmission route of mumps and related influencing factors, so as to provide a scientific basis for developing more effective prevention and control measures. Your child is invited to participate in this study. We sincerely hope that you will read the following carefully and decide whether to agree to participate according to your wishes.

I. Purpose of the study

To analyze the factors that may contribute to the spread of the disease by investigating the exposure history of mumps cases and their controls, and to provide a reference for public health policy.

II. Content of the study

Study subjects: confirmed mumps cases and their paired healthy control children under 15 years of age in Taizhou City.

Research method: Collecting relevant information through questionnaires, including but not limited to family environment, vaccination history, and contact history.

Study duration: approximately 20-30 minutes.

III. Benefits of Participating in the Study

Your participation will help to better understand the transmission mechanism of mumps and provide a scientific basis for protecting children's health.

IV. Possible Risks

This study involves only questionnaires and will not cause any harm to your or your child's health. All information will be kept strictly confidential and used only for scientific research.

V. Privacy Protection

Your personal information and the content of the questionnaire will be kept strictly confidential and only the research team will have access to it.

The results of the study will be presented in a summarized form and will not reveal any personally identifiable information.

Voluntary Principle

Your participation in the study is entirely voluntary. You have the right to withdraw from the study at any time without any repercussions.

Ethical Review

This study has been formally reviewed and approved by the Ethics Review Committee of Taizhou City Hospital of Integrative Medicine and Western Medicine (Len review number: 2023-ethics review LW-001, approval date: May 22, 2023).

VIII. Contact information

If you have any questions about the study, please contact:

Study leader: Tang Wanqin

Tel: 18112199399

Email: twqpinfan@163.com

IX. Informed Consent Statement

I have read and understood the above and voluntarily consent to my child's participation in this study.

Parent/Guardian Signature: _________________

Relationship to child: _________________

Date: _________________

Child's name: _________________

Child's date of birth: _________________

Thank you for your support and cooperation!

Taizhou Center for Disease Control and Prevention

Date: _________________
